# High Q-factor microring resonator wrapped by the curved waveguide

**DOI:** 10.1038/srep10078

**Published:** 2015-05-20

**Authors:** Dong-Po Cai, Jyun-Hong Lu, Chii-Chang Chen, Chien-Chieh Lee, Chu-En Lin, Ta-Jen Yen

**Affiliations:** 1 Department of Optics and Photonics, National Central University, 32001 Jhongli, Taiwan; 2 Optical Sciences Center, National Central University, 32001 Jhongli, Taiwan; 3 Department of Materials Science and Engineering, National Tsing Hua University, Hsinchu 30013, Taiwan

## Abstract

In this work, we study the performances of ring resonators of different type by analyzing the bending loss and the condition of the critical coupling. We propose that the bending loss of microring can be reduced by wrapping a concentrically curved waveguide. The difference of propagation constant between two concentrically curved waveguides can be tuned by adjusting the bus waveguide width to optimize the critical coupling. Furthermore, we propose to enlarge the difference of the propagation constant between two concentrically curved waveguides to maintain the circulating light in the ring to obtain higher quality factor. In this study, the highest quality factor that we measured is 7 × 10^5^.

The microring is an important and versatile integrated optics component. Several devices such as filter^1^, gyroscope^2^,^3^ and optical switch^4^ can be constructed by the microring. Besides, the microring can provide the ultra-high Q-factor to induce the “slow light” property to slow down the group velocity of propagating light^5^,^6^ and to improve the performance of gyroscope^2^,^3^. Over the past decade, several types of microring have been developed such as the single microring with single bus waveguide^7^,^8^ or with double bus waveguides^9^, the racetrack microring resonators^10^ and the multiple microrings^11^. The results reported in Ref. 2 and Ref. 3 show that the improvement of the Q-factor of microring can enhance the sensitivity of gyroscope. The microdisk and microring with the pulley-type coupling configuration have been reported for the applications of integrated optical isolators^12^,^13^ and sensor devices^14^. Recently, Cai *et al.* had reported the method to determine the coupling length and the propagation constant of the concentric waveguides^15^. This method is helpful to analyze the pulley-type microring.

In order to improve the Q-factor of microring, the maintenance of the light in the ring as long as possible (i.e, the reduction of the loss) is required due to the fact that Q-factor is inversely proportional to the decay constant of microring. Besides, the bending loss is one of the factors to mainly determine the performance of device for a microring structure. In the literatures, several guidelines in design, and cares during fabrication have been reported. For example, the sidewall roughness of the waveguides should be smoothed to decrease the scattering loss of waveguide^16^,^17^. For the racetrack microring, the increasing of the crosstalk between drop signal and add signal is critical^18^. The Q-factor can also be improved by increasing the radius of the microring to reduce the bending loss of the ring. By using the complex structure such as the multiple microrings^11^, the performance of microring resonator can also be improved.

In this work, we propose a design guideline to improve the performance of microring. Firstly, by wrapping the bus waveguide around the curved part of the microring, the bending loss of microring can be reduced. A longer curved bus waveguide to wrap the microring may provide higher Q-factor. Secondly, the large difference of propagation constant between the bus waveguide and microring, Δβ, (i.e. to reduce the light in the microring to couple toward the bus waveguide) is helpful to maintain the light in the ring. However, the condition of the critical coupling depends on Δβ^19^,^20^, and the increasing of Δβ should be finely tuned to optimize the critical coupling to improve the performance of microrings.

Several parameters of device including the ring waveguide width, bus waveguide width, radius of the ring and the gap width can be adjusted to enlarge Δβ. To facilitate the analyses between the different types of microring, we fix the circumference of the ring, the ring waveguide width and the gap width. The width of the bus waveguides is adjusted to reach the critical coupling condition.

## Results and Discussion

### The various microring resonators

**Figure 1:**
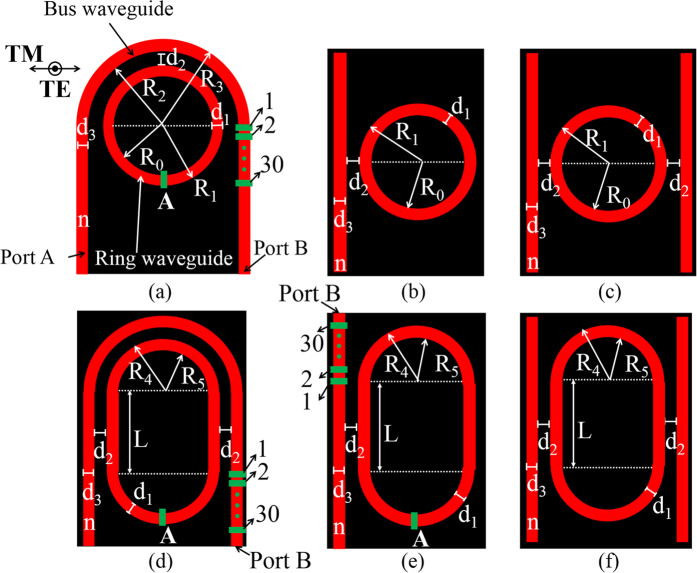
Schematic drawing of microring resonators. (**a**) shows pulley-type microring. (**b**) and (**c**) show microring with one bus waveguide and two bus waveguides, respectively. (**d**) shows pulley-type racetrack microring. (**e**) and (**f**) show racetrack microring with single bus waveguide and two bus waveguides, respectively. The green rectangles are the detectors to monitor the optical power.

The various microrings and the parameters are schematically shown in Fig. 1(a)-(f). Fig. 1(a) is a pulley-type microring. Fig. 1(b) are microrings with straight bus waveguide. Fig. 1 (d) are racetrack microrings. In Fig. 1(a)-(f), the circumference of the microrings is 27.835 μm for all cases. The inner radius, R_0_, and the outer radius, R_1_, of the microring are 4.33 μm and 4.53 μm in Fig. 1(a)-(c), respectively. For Fig. 1(a), the inner radius of the curved bus waveguide, R_2_, is 4.68 μm. The outer radius of the curved bus waveguide, R_3_, is varied from 4.83 μm to 4.93 μm. The corresponding width of the bus waveguide, d_3_, is varied from 0.15 μm to 0.25 μm. For Fig. 1(d)-(f), the circumference of the racetrack microring is defined as 2L + 2π[(R_4_ + R_5_)/2] to be 27.835 μm where L, R_4_ and R_5_ are the length of the straight waveguide, the outer and inner radii of the curved waveguide in the racetrack, respectively. Moreover, L is varied from 0.5 π μm to 2.5 π μm. For all cases (Fig. 1(a)-(f)), the width of the ring waveguide, d_1_, is 0.2 μm and the gap, d_2_, between the ring waveguide and the bus waveguide is chosen to be 0.15 μm. The refractive index of the silicon waveguide for the wavelength range from 1 μm to 2 μm is obtained from Ref. 21 to be around 3.48 and the refractive index of the background is unity. The index contrast, 
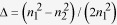
, for different wavelengths is around 0.46 where n_1_ is the index of waveguide and n_2_ is the index of cladding layer. The absorption of the material is neglected in this work.

In 2-D finite-difference time-domain method (FDTD) simulation, a monitor is positioned in the ring. A pulse light source at the central wavelength of 1.55 μm with TM polarization is launched to obtain the spectrum of the light stored in the ring. The corresponding bandwidth of pulse is around 400 nm. The TM polarization is defined as the direction of electric field is perpendicular to the top surface of device. In order to estimate the Q-factor, the impulse light at the resonance wavelength is launched into the structure.

### The Q-factors and the corresponding parameters for each microring resonator

For each resonator illustrated in Fig. 1, the difference of the propagation constant (Δβ) is changed by varying d_3_. We adopt the conformal transformation method^15^,^22^,^23^ and Helmholtz equation to calculate the propagation constants of the waveguides. From Ref. 15we concluded that the propagation constant of the outer curved waveguide is higher than that of the inner curved waveguide as the waveguide width is identical and the propagation constant of curved waveguide can be changed by adjusting the curved waveguide width. In Table 1, we list the higher and lower Q-factors for each resonator in Figs. 1 and the corresponding parameters. The larger Δβ between the bus waveguide and ring waveguide can be observed to be one of factors to obtain high Q-factor microing resonator. The results also show that the pulley-type microring [Fig. 1(a)] and the racetrack microring with one bus waveguide [Fig. 1(e)] can provide a higher Q-factor after adjusting the bus waveguide width.

### The bending loss of the microring resonators

**Figure 2:**
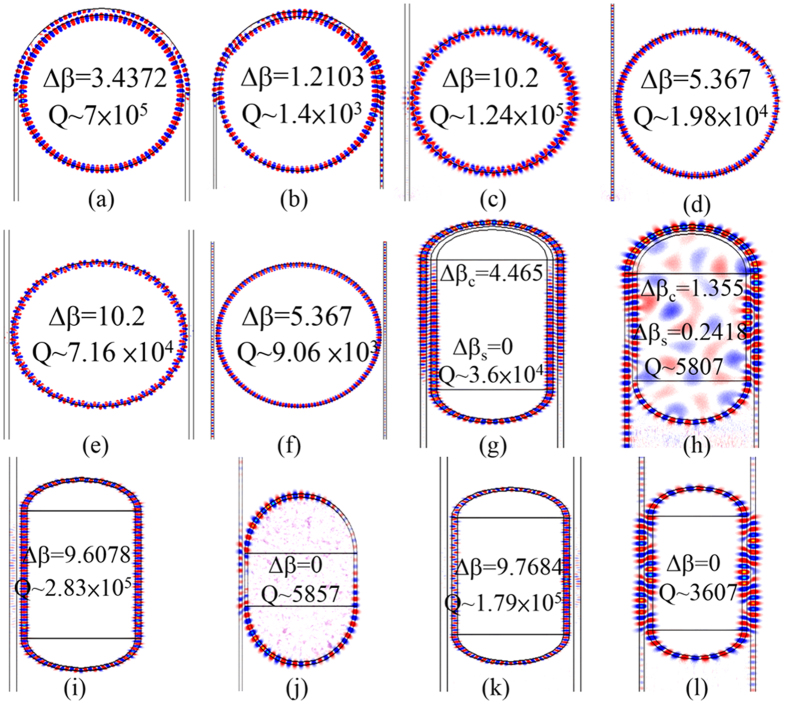
Normalized circulating field in the various microring resonators. For the identical type of microring resonator, the larger Δβ between bus waveguide and ring waveguide is helpful to obtain higher Q-factor. The corresponding resonance wavelengths and the widths of bus waveguide are listed in Table 1.

The electric fields of the microrings listed in Table 1 are shown in Figs. 2. The results show that the electric field in the bus waveguide of the higher Q-factor microrings [Fig. 2 (a)] is weaker than that in the lower Q-factor microrings [Fig. 2 (b)], respectively. According to the statements mentioned above, Δβ at the coupling region in the high Q-factor microring is larger than that in the low Q-factor microring. For high Q-factor microring, the propagating light stored in the ring is difficult to couple back to the bus waveguide. Additionally, the pulley-type microring resonator in Fig. 2 (a) can provide the highest Q-factor of 7 × 10^5^ due to the lower bending loss. The long interaction length of coupling region (concentrically curved waveguides) in the pulley-type microring can provide the lower bending loss than that of the other structures except the pulley-type racetrack microring [Fig. 1(d)].

The pulley-type racetrack microring as shown in Fig. 1(d) with the longest coupling region and large propagation constant difference between the curved waveguides, Δβ_c_, cannot obtain the higher Q-factor. It is due to the radius of the curved waveguide of the racetrack microring is much smaller than that of the circular microrings, which results in the larger bending loss from the curved bus waveguide.

**Figure 3:**
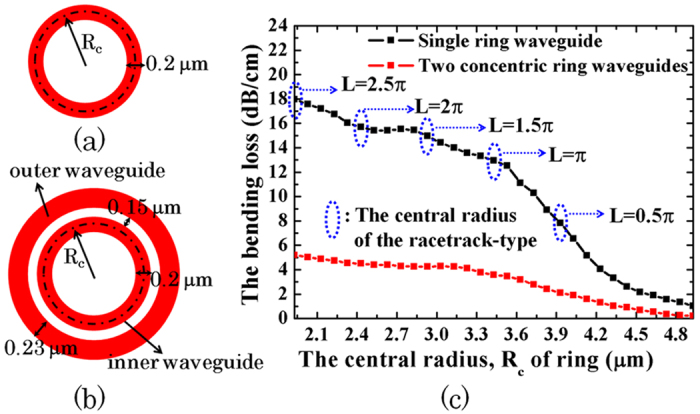
Bending loss. (**a**) and (**b**) show the schematic drawing of single ring waveguide and two concentric ring waveguides, respectively. (**c**) Bending loss per propagation distance of single ring waveguide and two concentric ring waveguides for different R_c_ are represented as the black line and red line, respectively.

In order to study the relation between the bending loss and the microring resonator wrapped by the curved waveguide, we calculate the bending loss of single curved waveguide and two concentrically curved waveguides. The single ring waveguide with the central radius, R_c_, defined as (R_0_ + R_1_)/2  =  4.43 μm is schematically shown in Fig. 3(a),(b) illustrates the two concentric ring waveguides. The gap between two ring waveguides is 0.15 μm. The inner and outer waveguide widths are 0.2 μm and 0.23 μm, respectively. We launch an impulse light into the ring and adopt the exponential decay fitting for the power detected in the ring to obtain the bending loss per propagation distance of ring waveguide by the method published in Ref. 7. Fig. 3(c) shows the bending loss per propagation distance of the single ring waveguide and the two concentric ring waveguides. We can observe that the bending loss of the two concentric ring waveguides is lower than that of the single ring waveguide, since the outer waveguide of the concentric ring waveguides can collect the light of the radiation loss of the inner waveguide and then couple the light back to the inner waveguide. Therefore, the bending loss of the ring can be reduced by wrapping curved bus waveguide.

For the microrings with the identical circumference, the central radius of the curved waveguide of the racetrack microring is smaller than that of the other circular microrings. Therefore, the total bending loss of racetrack microring resonator should be larger than the other type of microring resonator. [The total bending loss (dB) of the microrings is defined as bending loss (dB/cm) × length of curved waveguide (cm). In our study, the bending loss (dB/cm) is obtained from Fig. 3(c).] The total bending loss of the microring with the single and two bus waveguides is identical to be 7.3 × 10^−3^ dB. The total bending loss of the pulley-type microring is 5 × 10^−3^ dB. In Fig. 3(c), we also illustrate the bending loss per propagation distance of the curved waveguide in the racetrack microring for different L. The total bending loss of the racetrack microring with L of 0.5π, π, 1.5π, 2π and 2.5π can be obtained to be 1.93 × 10^−2^ dB, 2.93 × 10^−2^ dB, 2.75 × 10^−2^ dB, 2.4 × 10^−2^ dB and 2.18 × 10^−2^ dB, respectively. The total bending loss of the pulley-type racetrack microring with L of 0.5π, π, 1.5π, 2π and 2.5π can be obtained to be 1.24 × 10^−2^ dB, 1.87 × 10^−2^ dB, 1.79 × 10^−2^ dB, 1.57 × 10^−2^ dB and 1.43 × 10^−2^ dB, respectively. The results show the pulley-type microring can provide lower bending loss than other types of microring.

### The loss from output port

**Figure 4:**
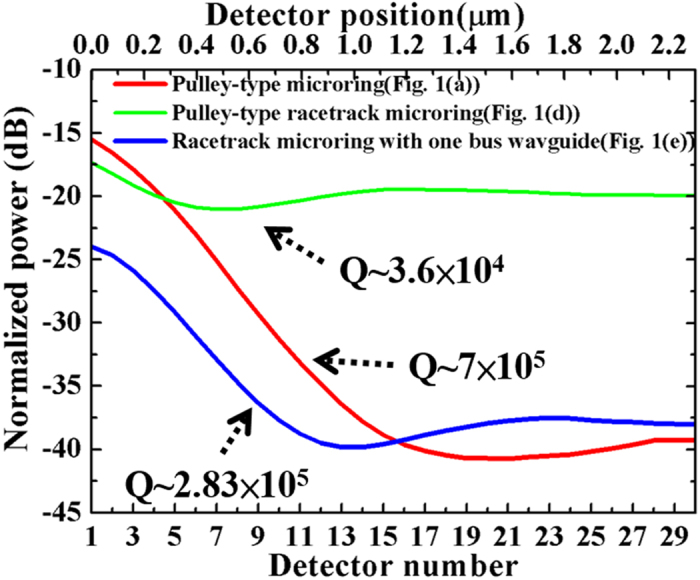
Power loss from output ports. The red, blue and green lines present the normalized power detected at Port B of the pulley-type microring resonator, the pulley-type racetrack microring resonator and the racetrack microring, respectively.

In order to investigate the power loss from Port B of the higher and lower Q-factor structures , we arrange thirty detectors at Port B as shown in Fig. 1(a),(d),(e). The distance between the detectors is around 80 nm. As the figures illustrated, the detector A is set to measure the power stored in the ring. The normalized power loss from Port B as shown in Fig. 4, which is defined as the power detected by the thirty detectors divided by the power detected by the detector A. The red line shows the normalized power losses for the pulley-type microring with d_3_ of 0.23 μm (Q = 7 × 10^5^). The blue line shows the normalized power losses for the racetrack microring as shown in Fig. 1(e) with d_3_ and L of 0.25 μm and 2.5π μm, respectively (Q = 2.83 × 10^5^). The decreasing of power as shown in Fig. 4 indicates that the circulating light in the bus waveguide at the output port is coupled to the ring waveguide. The output power measured at Detector 30 is almost the same to be around −40 dB, which indicates the fact that the critical coupling of the two structures is tuned to be almost identical. However, the Q-factor of the racetrack microring is lower than that of pulley-type microring with d_3_ of 0.23 μm, since the smaller bending radius of the curved waveguide in the racetrack microring provides the higher bending loss than that of the pulley-type microring.

From the result in Fig. 3(c), we can conclude that the bending loss of pulley-type racetrack microring is lower than that of the racetrack microring. However, the Q-factor of the pulley-type racetrack microring is lower than that of racetrack microring. The green line in Fig. 4 shows the normalized power losses for the pulley-type racetrack microring with d_3_ and L to be 0.2 μm and 2.5π μm, respectively (Q = 3.6 × 10^4^). We can observe that the measured power changes slightly from Detector 1 to Detector 30. The result indicates that the energy of the resonator losses from the output port. In this case, the critical coupling may not be as good as that in Fig. 2(a) because the larger energy loss at the output port. From another viewpoint, among all structures that we analyze in this work, only the coupling region of the pulley-type racetrack microring consists of the straight waveguides and the curved waveguides. In this structure, the device parameters include the ring waveguide width, bus waveguide width, and radius of the ring and the gap width. The change of the parameters will individually change the coupling coefficient of the straight waveguides but also that of the curved waveguides. Therefore, the critical coupling of the pulley-type racetrack microring might be very difficult to be optimized.

In this work, we propose the guidelines to design the high-Q microring. Firstly, by wrapping the curved waveguide around the microring to form the pulley coupler, the bending loss can be effectively decreased. The longer interaction length of coupling region such as the concentrically curved waveguides is helpful to decrease the radiation loss and improve the Q-factor of the resonator. Secondly, the large difference of propagation constant, Δβ, between the microring and the bus waveguide can maintain better the propagating light in the mcroring. Thirdly, the critical coupling can be rapidly optimized by adjusting the bus waveguide width to increase the Q-factor. The reasons mentioned above and the obtained results by comparing the performance of the different type ring resonators show that the pulley-type microring may provide higher Q-factor.

## Method

Since the 3D-FDTD method is very time-consuming, in this work, we adopt the 2D FDTD method to investigate the in-plane energy decay in the microring^1^,^24^.

### Q-factor

The Q-factor is calculated by monitoring the energy decay constant in the microring and exponential decay fitting. For the microring with the resonant wavelength at λ, the Q-factor is defined as
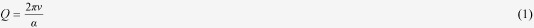
where ν and α are the frequency of light and the decay constant of stored energy, respectively.

### The conformal transformation^15^,^22^,^23^ method and Helmholtz equation

The conformal transformation is the transformation preserved the local angle in the complex plane, w-plane. The analytic function in w-plane w = f(z) cannot be conformal as the derivative of f(z) = 0 or ∞. In the conformal mapping, the analytic function w = f(z) where w = u + iv and z = x + iy is employed to transform the structure of device from the original coordinate, z-plane, to a new coordinate, w-plane.

The conformal transformation method is suitably adopted to analyze the curved waveguide. The conformal transformation can transform the complex structure of curved waveguide to the simple structure of straight waveguide in such a way that
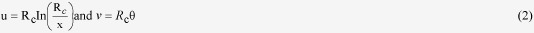
where u and v is the new coordinate after the transformation. R_c_ is the central radius of curved waveguide. x and y are respectively the coordinate and the angle before the transformation.

To obtain the propagation constant of curved waveguide, the wave equation is solved after the conformal transformation. Before the conformal transformation, the two dimensional scalar wave equation can be represented as
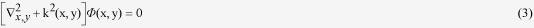
The solution of equation (3) in new coordinate u, v can be defined by original coordinate x, y with the relation
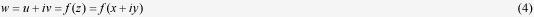
where f is the analytic function. The Cauchy-Riemann equation and Laplace’s equation are expressed as equation (5) and equation (6), respectively.
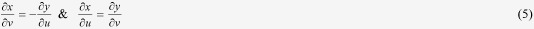

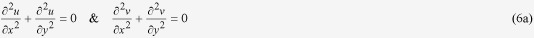

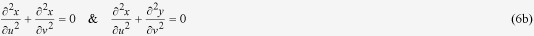


Expending the term of 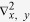 in equation (3) by using equation (5) and equation (6a), the scalar wave equation can be rewritten as
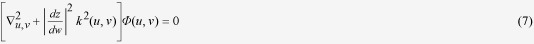
where
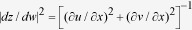
By using the conformal transformation, w in equation (4) can be defined as
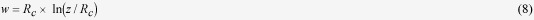
for which
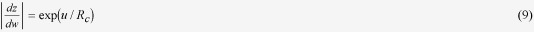
After the conformal transformation, the curved waveguide can be regarded as the straight waveguide. By separating the wave function *Φ*(*u, v*) into two functions:
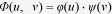
Equation (7) can be rewritten as the one-dimensional Helmholtz equation as
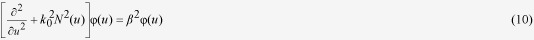
where β is the propagation constant defined as *k*_0_ n_eff_. The effective index is n_eff_. The ϕ(*u*) is the electric field distribution in w-plane. N(*u*) defined as n_˙_exp(*u*/R_c_) is the refractive index distribution in w-plane. After the conformal transformation, the step profile of refractive index can be transformed to the exponential profile of refractive index. By solving equation (10) as the eigen-problem, the effective indices (eigenvalues) and the corresponding electric fields (eigenvectors) can be obtained.

### The bending loss

The propagation loss of curved waveguide can be expressed as
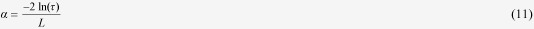
where τ is the attenuation of the circulating light per around trip. L is the length of the microring.

## Author Contributions

D.P.C., J.H.L., C.C.C., C.C.L., C.E.L. and T.J.Y. designed and analyzed data. D.P.C. and J.H.L. executed the simulations. D.P.C., C.C.C., C.C.L., C.E.L. and T.J.Y. wrote the manuscript text. All authors reviewed the manuscript.

## Additional Information

**How to cite this article**: Cai, D.-P. *et al*. High Q-factor microring resonator wrapped by the curved waveguide. *Sci. Rep.* **5**, 10078; doi: 10.1038/srep10078 (2015).

## Figures and Tables

**Table 1:** Higher and lower Q-factors of each type of microring and the corresponding parameters.
